# A Rare Variant of *ANK3* Is Associated With Intracranial Aneurysm

**DOI:** 10.3389/fneur.2021.672570

**Published:** 2021-06-25

**Authors:** Junyu Liu, Xin Liao, Jilin Zhou, Bingyang Li, Lu Xu, Songlin Liu, Yifeng Li, Dun Yuan, Chongyu Hu, Weixi Jiang, Junxia Yan

**Affiliations:** ^1^Department of Neurosurgery, Xiangya Hospital, Central South University, Changsha, China; ^2^Department of Epidemiology and Health Statistics, Xiangya School of Public Health, Central South University, Changsha, China; ^3^The People's Hospital of Guangxi Zhuang Autonomous Region, Nanning, China; ^4^Department of Neurology, Hunan People's Hospital, Changsha, China; ^5^Hunan Provincial Key Laboratory of Clinical Epidemiology, Xiangya School of Public Health, Central South University, Changsha, China

**Keywords:** intracranial aneurysm, *ANK3*, candidate gene, neurosurgery, whole exome sequencing

## Abstract

Intracranial aneurysm (IA) is a cerebrovascular disorder in which abnormal dilation of a blood vessel results from weakening of the blood vessel wall. The aneurysm may rupture, leading to subarachnoid hemorrhage with severe outcomes. This study was conducted to identify the genetic factors involved in the etiology of IA. Whole-exome sequencing was performed in three IA-aggregate families to identify candidate variants. Further association studies of candidate variants were performed among sporadic cases and controls. Bioinformatic analysis was used to predict the functions of candidate genes and variants. Twenty variants were identified after whole-exome sequencing, among which eight were selected for replicative association studies. *ANK3* c.4403G>A (p.R1468H) was significantly associated with IA (odds ratio 4.77; 95% confidence interval 1.94–11.67; *p*-value = 0.00019). Amino acid R1468 in *ANK3* was predicted to be located in the spectrin-binding domain of ankyrin-G and may regulate the migration of vascular endothelial cells and affect cell–cell junctions. Therefore, the variation p.R1468H may cause weakening of the artery walls, thereby accelerating the formation of IA. Thus, *ANK3* is a candidate gene highly related to IA.

## Introduction

Intracranial aneurysm (IA) is a cerebrovascular disorder causing structural defects in the middle muscular layer of the artery. Its prevalence is estimated to be around 1–5% (1) worldwide and around 7% of adults aged 35–75 years in China (2). Although patients are typically asymptomatic, the result of aneurysmal subarachnoid hemorrhage (SAH) caused by the rupture of IA is crippling and life-threatening (3). Multiple known risk factors including cigarette smoking, alcoholism, and hypertension are involved in the formation and rupture of IA (4). The aggregation of patients in IA families suggests that genetic factors contribute to disease susceptibility (5). Family-based genome-wide linkage studies located IA susceptible loci in chromosomal regions such as 1p34.3–36.13, 7q11, and 19q13 (6). With the development of next-generation sequencing techniques in recent years, a series of genes such as *ADAMTS15, RNF213, PCNT, THSD1, ARHGEF17*, and *LOXL2* have been identified as potential genetic factors causing familial IA (7–12). However, the findings of studies between different populations show little overlap, illustrating the significant genetic heterogeneity of IA.

As low-frequency and rare variants [minor allele frequency (MAF) < 0.05] are likely associated with a higher risk of IA pathogenesis, we performed whole-exome sequencing (WES) in three Chinese IA families and replicated several candidate variants in sporadic cases and controls to extend the results to a broad disease phenotype. Furthermore, we downloaded five Gene Expression Omnibus (GEO) datasets and performed bioinformatic analysis to evaluate the expression levels and functions of the candidate genes.

## Materials and Methods

### Study Population

**Figure 1:**
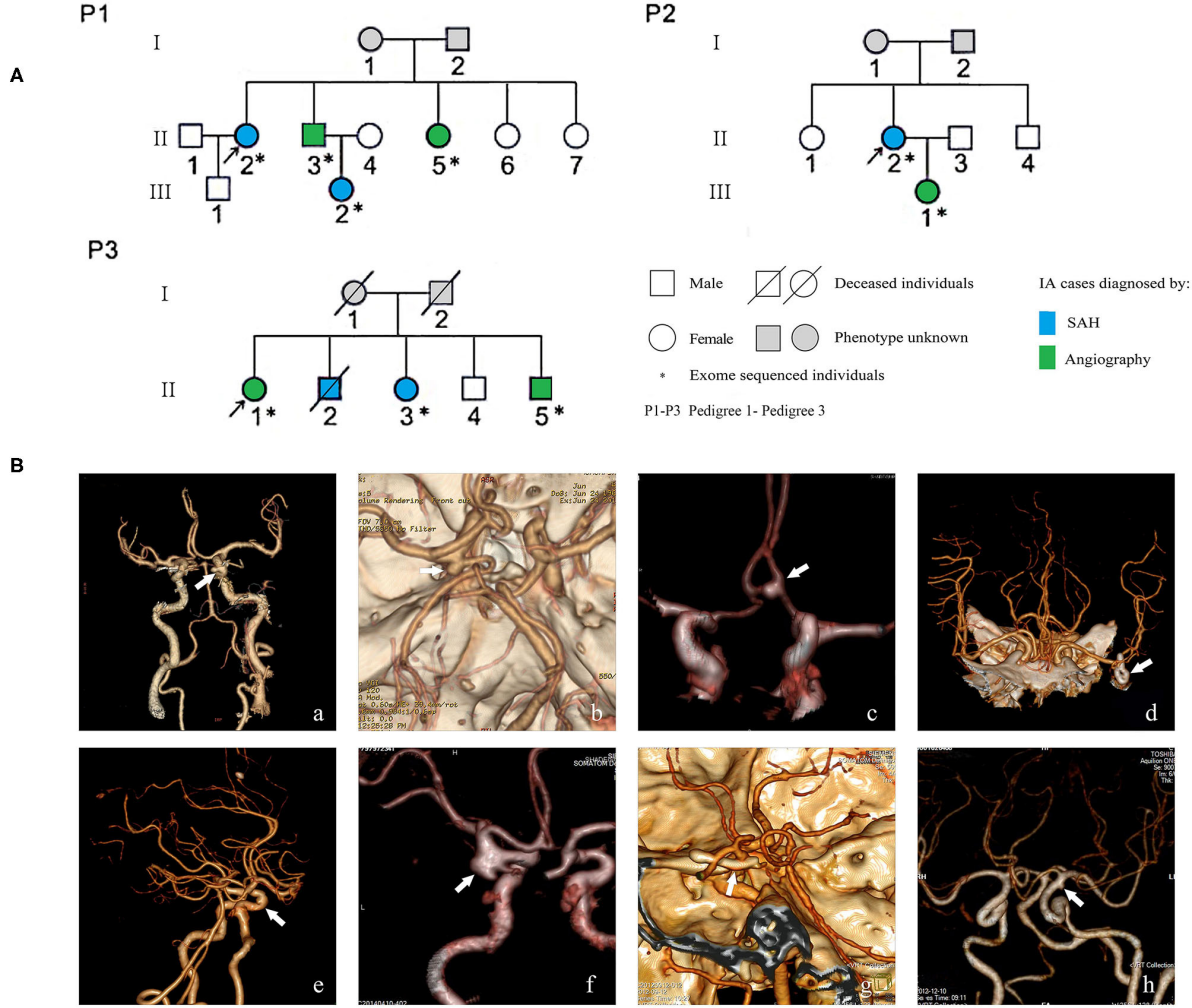
Pedigrees of three Chinese intracranial aneurysm (IA) families **(A)** and angiographic images of eight familial IA patients **(B)**. SAH indicates subarachnoid hemorrhage; white arrows in the images of computer tomography angiography point to the locations of IAs. The digital image of patient P1-II-3 was not available because the patient was transferred to Xiangya Hospital; beforehand, his examination and surgery, which confirmed his diagnosis of IA, were performed in a different hospital.

A cohort of familial and sporadic IA cases was recruited from the Department of Neurosurgery and Neurology in Xiangya Hospital and Hunan People's Hospital from January 2016 to December 2019. Both familial and sporadic cases were diagnosed by magnetic resonance angiography, computed tomography angiography, or digital subtraction angiography. Angiography image results were interpreted by at least a radiologist and neurosurgeon independently. Cases of disagreement were resolved by consensus or interpretation by a third physician. Several patients with SAH were confirmed by intracranial surgery performed as an emergency operation but with suspicious computed tomography angiography imaging. Patients with IA diagnosed with other vascular diseases such as cerebral arteriovenous malformation, moyamoya disease, or several hereditary diseases including hereditary hemorrhagic telangiectasia, Marfan's syndrome, or Sturge-Weber syndrome, were excluded. Individual demographic information and lifestyle data were collected through an interview conducted by the clinical reception staff. Clinical information was collected by consulting the medical record system. Peripheral blood samples were collected from all enrolled individuals. For familial IA, each family has two or more patients with IA in the first- to third-degree relatives. Three families with nine confirmed IA cases were selected for WES (Figure 1). A total of 384 sporadic IA cases and 384 controls was used for further association studies of the selected candidate variants. Control individuals were recruited from the community health service center in the same geographic region of Hunan Province, China, during the same period. The controls had undergone an annual health check-up and structured interview and showed no medical or family history of IA, SAH, or any other known cerebrovascular disease (13). This study was approved by the Medical Ethics Committee of Clinical Pharmacology Institute, Central South University, China (CTXY-150002-1), and written informed consent was obtained from all participants.

### Whole Exome Sequencing

Genomic DNA was extracted from the peripheral blood of patients with IA and control individuals using TIANamp Blood DNA Extraction Kits (TIANGEN Biotech Co., Ltd., Beijing, China). WES was performed by Novel Bioinformatics Co., Ltd. (Shanghai, China). The exome was captured with an Agilent Sureselect All Exon V6 Kit (Agilent Technologies, Santa Clara, CA, USA) and sequenced on a HiSeq X Ten platform (Illumina, San Diego, CA, USA). Sequence mapping and variant detection were performed using the Burrows-Wheeler Aligner tool and Genome Analysis Toolkit software, respectively.

To prioritize the variants, a series of filters was used. Variants were given higher priority if they were (1) predicted to affect the protein-coding sequences including non-synonymous single-nucleotide variations such as missense, nonsense, or splice site variations, and insertions or deletions in the consensus coding sequencing region; (2) damaging or unknown as predicted by protein prediction programs [Sorting Intolerant From Tolerant (SIFT) and Polymorphism Phenotyping V2 (PolyPhen-2)]; (3) less common in reference databases (MAF < 0.05 in East Asian population according to the 1,000 genome database (http://www.1000genomes.org/) and gnomAD (https://gnomad.broadinstitute.org/); (4) high-quality variants (sequence read depth was more than 8x); (5) shared by all affected individuals in more than one family.

### Sanger Sequencing

To confirm the variants discovered by WES, Sanger sequencing was conducted in the patients of three families. Primers (Supplementary Table 1) were designed according to NCBI (http://www.ncbi.nlm.nih.gov/). After polymerase chain reactions, the sequencing was performed using a BigDye Terminator v3.1 Cycle Sequencing Kit (Applied Biosystems, Foster City, CA, USA) on an ABI GeneAmp 3730xl DNA Sequencer (Applied Biosystems).

### Venn Analysis of GEO Databases

For gene expression analysis, we downloaded five databases (Supplementary Table 2) from the GEO (https://www.ncbi.nlm.nih.gov/gds/) under the accession number GSE 13353 [11 ruptured IA (RIA) and 8 unruptured IA (UIA)], GSE 15629 (8 RIA and 6 UIA), GSE26969 (3 UIA), GSE54083 (8 RIA and 5 UIA) and GSE66238 (7 UIA) (14–18), and the superficial temporal arteries or meningeal media arteries were isolated as control arteries. We compared gene expression between ruptured and unruptured IA wall samples as well as unruptured IA and control samples. Gene expression studies were performed in Finnish, Polish, Chinese, Japanese, and Lebanese populations and reperformed using the limma package. The threshold for identification of differentially expressed genes was set as *p* < 0.05 and |log2 fold-change (FC)| > 1.0. Next, Venn analysis was carried out between the genes of the filtered variants and differentially expressed genes from the five online databases to explore the shared genes, which was checked in online Venn software (http://www.ehbio.com/test/venn/#/). Genes with log2FC < 0 were considered as down-regulated, whereas those with log2FC > 0 were considered as up-regulated.

### Replicate Association Study in Sporadic Cases

Candidate variants for the association study were selected based on (1) the commonness of the variants: the allele frequency of a variant identified in familial patients; (2) the results of Venn analysis: the overlapped variants in WES filtered candidate list and differently expressed genes in IA specimens from the GEO databases, and (3) the functional relevance of IA: genes whose function was associated with current known IA pathology such as inflammation, extracellular matrix composition, angiogenesis, and vascular remodeling (19). For variants in overlapped genes in the WES filtered database and GEO differently expressed gene databasets as well as the functional relevance variants, more common variants were given more priority in the replicated association study. Genotyping of the candidate variants was performed using the Sequenom MassARRAY platform (Agena Bioscience, Inc., San Diego, CA, USA). Primers were designed using ASSAY DESIGN SUITE V2.0 based on a single-nucleotide polymorphism (SNP) locus (http://agenacx.com, Agena Bioscience, Inc). PCR amplification was performed on an ABI GeneAmp 9700 384-well Dual (Applied Biosystems), and SNP alleles were identified by the different masses of the extended prime primers using matrix-assisted laser desorption/ionization–time of flight mass spectrometry on MassARRAY Nanodispenser RS1000 (Sequenom, Inc., San Diego, CA, USA). The mass spectrometry peaks were detected using Typer 4.0 software (Agena Bioscience, Inc.) and the genotypes of each sample target locus were interpreted based on the mass spectrum peak map. Genotyping was performed by an operator blinded to the sample status (case or control subjects). The primers listed in Supplementary Table 3 were designed using Assay Design 3.1 (Sequenom, Inc.).

### Homology Alignment and Functional Analysis

Homology of the candidate variants was determined using protein BLAST (http://blast.ncbi.nlm.nih.gov/Blast.cgi). Additional functional analysis of the candidate genes was performed using bioinformatic methods. STRING database (https://string-db.org/) was used to generate a protein–protein interaction network and a computational model was created using SWISS MODEL (https://swissmodel.expasy.org/) to predict the effects of variations on domain structure.

### Statistical Analysis

For mRNA expression analysis, GEO 2R was used to compare the differences in the mRNA levels between different groups. Genes with both *p*-value < 0.05 and |log2FC| > 1.0 were considered as potentially differentially expressed. For the selected candidate variants, their association with IA was analyzed using SPSS 21.0 software (SPSS, Inc., Chicago, IL, USA). Clinical characteristics of the participants are presented as the mean ± standard deviation for normally distributed continuous variables and proportions for categorical variables. Student's *t*-test, chi-square test, or Fisher's exact test were used to compare differences between the groups for continuous and categorical variables. Multivariate logistic regression analysis was used to estimate the odd ratio and 95% confidence interval of each SNP after adjusting for known risk factors. Bonferroni correction based on 20 independent effective tests was used to adjust the significance level of the association (0.05/20 = 0.0025). A *p*-value < 0.0025 was considered as a significant association.

## Results

### Characteristics of Study Participants

This study included 9 familial IA participants, 384 sporadic IA participants, and 384 control individuals. Female sex was predominant in all three groups (88.89%, 69.53%, and 69.53%, respectively). The mean age of the familial IA cases was lower than that of the sporadic cases (50.50 ± 13.11 vs. and 57.10 ± 10.60 years) and the mean age of normal controls (66.54 ± 2.12 years) was higher than that of the other two groups. Four (44.44%) familial and 198 (51.56%) patients with sporadic IA suffered from a history of SAH. Smoking, drinking, medical history of diabetes, and hyperlipemia were more frequent in the control group. The detailed characteristics and clinical information of the study participants are summarized in Table 1.

**Table 1:** Clinical and demographic characteristics of study participants.

	**Exome sequencing of 3 families**	**Sporadic IA cases**	**Controls**	**Sporadic IA vs. control**
				***p*-value**
*N*	9	384	384	–
Female, *n* (%)	8 (88.89)	267 (69.53)	267 (69.53)	–
Age, years				
Mean ± SD	50.50 ± 13.11	57.10 ± 10.60	66.54 ± 2.12	<0.01
Range	29–67	22–88	53–70	–
SAH, *n* (%)	4 (44.44)	198 (51.56)	–	–
Hypertension, *n* (%)	6 (66.67)	208 (54.17)	200 (52.08)	0.56
Diabetes, *n* (%)	1 (11.11)	24 (6.25)	65 (24.74)	<0.01
Hyperlipemia, *n* (%)	4 (44.44)	148 (38.54)	213 (55.47)	0.04
Smokers, *n* (%)	1 (11.11)	45 (11.72)	92 (23.96)	<0.01
Drinkers, *n* (%)	2 (22.22)	16 (4.17)	77 (20.05)	<0.01
Single IA, *n* (%)	8 (88.89)	248 (63.58)	–	–
Location of IA, *n* (%)				
Internal carotid artery	1 (11.11)	151 (39.32)	–	–
Middle cerebral artery	1 (11.11)	74 (19.27)	–	–
Anterior cerebral artery	0 (0)	23 (5.99)	–	–
Anterior communicating artery	1 (11.11)	43 (11.20)	–	–
Posterior communicating artery	6 (66.67)	55 (14.32)	–	–
Vertebral/basilar artery	0 (0)	21 (5.47)	–	–
Posterior cerebral artery	0 (0)	11 (2.87)	–	–
Others	0 (0)	6 (1.56)	–	–

–*, not available*.

### Whole Exome Sequencing Analysis

WES generated 9.76–14.42 Gb bases of raw data for the nine familial participants, of which 9.47–13.23 Gb bases could be effectively mapped to the genome. The mean sequencing depth was between 90.3 and 125X. The average percentage of exomes covered into a read depth over 8X was 98.4%. Single-nucleotide variants were found between 653,710 and 811,492, whereas insertions and deletions were present between 82,537 and 105,085 (Supplementary Table 4).

We focused on non-synonymous single-nucleotide variants and frameshift insertions/deletions in exonic/splice regions, which were present in individuals with IA in more than one family. After filtering of low-frequency or rare variants (illustrated in Table 2), 22 variants remained.

**Table 2:** Variant filtration steps of whole exome sequencing.

**Filtration steps**	**Number of variants**
1. Non-synonymous single-nucleotide variants/frameshift deletion/stop gain/stop lost located in exonic/splicing region	20,305
2. Variants judged as SIFT prediction = damaging/unknown and polyphen-2 prediction = possibly damaging/probably damaging/unknown	4,254
3. MAF < 0.05 in 1 KGP (East Asian population) and gnomAD Database (East Asian population)	3,635
4. Variants with sequenced base depth > 8x	3,442
5. Variants shared in more than 1 family	22

Sanger sequencing was performed to confirm the exome findings. Except for c.1249C>T (p.R417Ter) in *ANKRD36C* (rs76474100) and c.268G>A (p.G90S) in *PSPH* (rs75395437), 20 variants were validated in the corresponding individuals. The detailed information is listed in Table 3. Among the variants, *GUCY1B3* c.17+5T>G, *ADAD2* c.1298+6C>G, and *PNMT* c.411-3C>T were in the splice region, and the remaining were missense and frameshift variants in the exonic region.

**Table 3:** Filtered variants shared in familial IA cases.

**Allele acount in 9 cases**	**Chr_position**	**Gene**	**Function**	**rs_number (dbSNP135)**	**Ensembl number**	**Nucleotide change**	**Amino acid change**	**MAF in 1KGP /gnomAD Database (East Asian population)**	**Segregated family**	**Partially carried cases**
7	1_153604293	*S100A1*	MS	rs1046256	ENST00000292169	c.261C>G	p.N87K	0.0079/0.01304	P2	P1(II-2,3,5), P3(II-1,5)
6	1_3410410	*MEGF6*	MS	rs61910697	ENST00000356575	c.4312G>A	p.G1438R	0.0476/0.04943	P1,P2	–
6	10_26998637	*PDSS1*	MS	rs77826284	ENST00000376203	c.407T>G	p.F136C	0.0228/0.02279	P3	P1(II-2,3,5)
4	10_61840324	*ANK3*	MS	rs74777754	ENST00000280772	c.4403G>A	p.R1468H	0.0139/0.01415	P3	P1(II-3)
4	14_61512802	*SLC38A6*	MS	rs117560154	ENST00000267488	c.842T>C	p.M281T	0.0258/0.02149	P2	P3(II-1,3)
4	19_45648915	*PPP1R37*	MS	rs539710409	ENST00000421905	c.1589C>T	p.P530L	0.003/0.0101	P3	P1(II-2)
4	21_33706638	*URB1*	MS	rs148134142	ENST00000382751	c.4691T>C	p.L1564P	0.0417/0.03852	P2	P3(II-1,3)
4	3_195452018	*MUC20*	MS	rs2688539	ENST00000445522	c.439A>G	p.S147G	0.001/0.04539	P3	P2(II-2)
4	4_674350	*MYL5*	MS	rs376244258	ENST00000400159	c.345delC	p.D115EfsTer15	0.006/0.004584	P2	P3(II-1,5)
4	4_38972691	*TMEM156*	MS	rs140693293	ENST00000381938	c.890A>G	p.Ter297=	0.0198/0.01965	P2	P1(II-2,5)
4	4_156693910	*GUCY1B3*	MS	rs76851701	ENST00000505764	c.17+5T>G	-	0.0308/0.04044	P3	P1(II-3)
4	6_159646624	*FNDC1*	MS	rs117546892	ENST00000297267	c.942G>T	p.Q314H	0.0139/0.01542	P2	P3(II-1,5)
4	7_39611992	*YAE1D1*	MS	rs79951226	ENST00000223273	c.368C>T	p.S123L	0.0139/0.01513	P2	P3(II-1,3)
4	19_48049175	*ZNF541*	MS	rs140680651	ENST00000314121	c.611G>C	p.S204T	0.0446/0.03816	P3	P2(III-1)
4	7_64169017	*ZNF107*	MS	rs375319415	ENST00000344930	c.2335_2336insA	p.Y780VfsTer54	0.001/0.00637	P2	P1(II-2)
3	1_247582310	*NLRP3*	MS	rs117287351	ENST00000336119	c.214G>A	p.V72M	0.0109/0.00922	P2	P1(II-2)
3	16_84229309	*ADAD2*	MS	rs191155110	ENST00000268624	c.1298+6C>G	–	0.0397/0.04097	P2	P1(III-2)
3	17_37826201	*PNMT*	MS	rs60871117	ENST00000269582	c.411-3C>T	–	0.0357/0.03501	P2	P3(II-5)
3	19_58992001	*ZNF446*	MS	rs58632700	ENST00000594369	c.1261C>T	p.R421W	0.0119/0.01301	P2	P1(II-5)
3	4_77025779	*ART3*	MS	rs143599971	ENST00000355810	c.1000C>T	p.P334S	0.0119/0.01455	P2	P1(III-2)

*MS, missense; MAF, minor allele frequency; P1, pedigree 1 (according to Figure 1); P2, pedigree 2; P3, pedigree 3*.

### Gene Expression Study Based on Public Database

**Figure 2:**
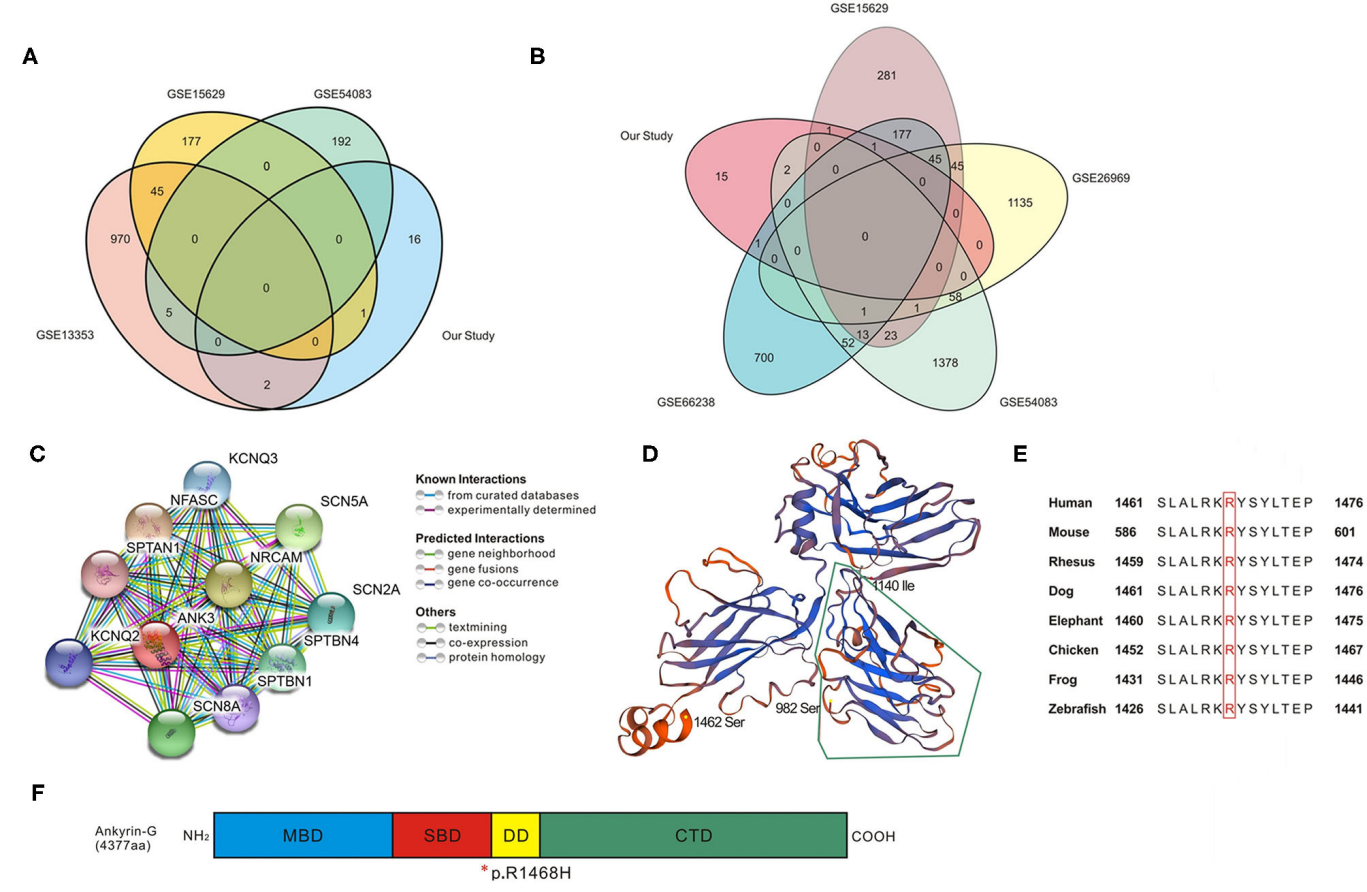
Bioinformatic analysis for candidate genes. **(A,B)** Venn analysis in UIA vs. RIA groups and UIA vs. Control groups; details of overlapped genes are shown in Table 4. **(C)** Protein–protein interaction network analyzed using STRING database. The network codes represent the proteins produced by corresponding protein-coding genes which are mainly associated with ion channels. **(D)** Computational model of SBD domain (982–1,462 amino acids, seq identity 77.28%). The segment surrounded by green lines (982–1,140 amino acids) is critical in bridging spectrin/actin cytoskeleton. **(E)** Conservation alignment showing that the mutated amino acids of ANK3 are conserved among different species. **(F)** Schematic diagram depicting the domain structure of Ankyrin-G (4,377 amino acids in human). The membrane binding domain (MBD, colored in blue) is comprised of 24 ANK repeats folded into a super-helical structure. The spectrin-binding domain (SBD, colored in red) can coordinate integral membrane proteins to the membrane skeleton, and the variant discovered in our study is at the end of this domain. The death domain (DD, colored in yellow) is highly conserved and the C-terminal domain (CTD, colored in green) is the most variable region to modulate the affinity and cellular localization of binding partners with ankyrin. *, the location of p.R1468H in the end of SBD domain.

The IA wall samples and control STA samples in five GEO databases were divided into UIA vs. RIA and UIA vs. control groups. After analysis, 1,022, 223, and 198 genes showed significant differential expression within the UIA vs. RIA group in GSE13353, GSE15629, and GSE54083, respectively. A total of 588, 1,321, 1,533, and 1,026 genes was differentially expressed between UIA and control samples in GSE15629, GSE26969, GSE54083, and GSE66238, respectively. Venn analysis was performed to identify the shared candidate variants with the five GEO databases (Figures 2A,B), and five genes were confirmed to be differentially expressed among RIA and UIA tissues and control vessels. *ANK3, GUCY1B3*, and *FNDC1* were shared in two databases (GSE13353&GSE54083, GSE13353&GSE15629, GSE15629&GSE66238, respectively), and were up-regulated in UIA compared to in RIA or controls. *SLC38A6* and *ART3* were in the GSE54083 and GSE66238 databases, respectively. The expression of *SLC38A6* and *ART3* was decreased in UIA compared to in controls, but increased expression of *SLC38A6* was identified in UIA rather than in RIA. The details are listed in Table 4.

**Table 4:** Venn analysis of differently expressed genes shared in GEO Databases and our study.

**Genes**	**UIA vs. RIA**				**UIA vs. Control**			
	***p*-value**	**log2FC**	**GEO Number**	**Style**	***p*-value**	**log2FC**	**GEO Number**	**Style**
*ANK3*	0.00035	1.05	GSE13353	Up	0.00061	1.35	GSE54083	Up
*SLC38A6*	0.0083	1.07	GSE54083	Up	0.000000047	1.40	GSE54083	Up
*GUCY1B3*	0.00061	1.57	GSE13353	Up	0.000081	−1.31	GSE15629	Down
*FNDC1*	0.0082	1.03	GSE15629	Up	0.0000079/0.000050	1.87/3.56	GSE15629/GSE66238	Up/up
*ART3*	–	–	–	–	0.00014	−4.31	GSE66238	Down

### Replicate Association Study of Several Candidate Variants

In the candidate list, variants in genes that were differentially expressed in IA tissues (*ANK3, GUCY1B3, FNDC1, SLC38A6*, and *ART3*) or those that potentially had relevance to IA biology (*S100A1, PDSS1*, and *MEGF6*) were prioritized in the replicate association studies. Previous studies showed that *S100A1* may function in vascular endothelium maintenance (20, 21), and *PDSS1* (22) and *MEGF6* (23) play roles in extracellular matrix integrity. Thus, variants in these eight genes were genotyped, and the results of the association study are summarized in Table 5.

**Table 5:** Replication and association study of sporadic intracranial aneurysms and controls.

**Gene**	**rs_number (dbSNP135)**	**Ensembl number**	**Nucleotide change**	**Amino acid change**	**Sporadic cases**			**Controls**			**OR(95%CI)**	***p*-value**
					**Ref/Ref**	**Ref/Alt**	**Alt/Alt**	**Ref/Ref**	**Ref/Alt**	**Alt/Alt**		
*S100A1*	rs1046256	ENST00000292169	c.261C>G	p.N87K	367	17	0	370	14	0	1.22 (0.60–2.52)	0.582
*PDSS1*	rs77826284	ENST00000376203	c.407T>G	p.F136C	359	24	1	360	24	0	1.04 (0.59–1.86)	0.883
*MEGF6*	rs61910697	ENST00000356575	c.4312C>T	p.G1438R	351	32	1	347	36	1	0.88 (0.54–1.44)	0.616
*ANK3*	rs74777754	ENST00000280772	c.4403G>A	p.R1468H	357	27	0	378	6	0	4.77 (1.94–11.67)	0.00019
*GUCY1B3*	rs76851701	ENST00000505764	c.17+5T>G	–	371	13	0	357	27	0	0.46 (0.24–0.91)	0.023
*FNDC1*	rs117546892	ENST00000297267	c.942G>T	p.Q314H	372	12	0	377	7	0	1.74 (0.68–4.46)	0.245
*SLC38A6*	rs117560154	ENST00000267488	c.842T>C	p.M281T	369	14	1	371	12	1	1.16 (0.54–2.47)	0.700
*ART3*	rs143599971	ENST00000355810	c.1000C>T	p.P334S	372	12	0	369	13	2	0.79 (0.37–1.72)	0.557

*Ref indicates reference allele; Alt, alternative allele; OR, odds ratio; 95%CI, 95% confidence interval*.

Our results showed that the variant of *ANK3* c.4403G>A (p.R1468H) was significantly associated with the risk of sporadic IA after Bonferroni correction [odds ratio (OR), 4.77; 95% confidence interval (95% CI), 1.94–11.67; *p*-value, 0.00019]. The splice site variant of *GUCY1B3* c.17+5T>G showed a lower MAF in sporadic cases compared to in the control population and may have protective effects in IA (OR, 0.46; 95% CI, 0.24–0.91; *p*-value, 0.023). However, this association did not reach the adjusted significance level (0.0025). No associations were observed for the other six variants (all *p* > 0.05).

### Function Prediction for *ANK3* and Its Variant

The *ANK3*-centered protein-protein interaction network generated from the STRING database is shown in Figure 2C. Through literature survey, it was found that *ANK3* encodes the protein ankyrin-G, which is expressed in various tissues including the kidney, brain, heart, and thyroid. The predicted domains of ankyrin-G contain a membrane-binding domain (MBD), spectrin-binding domain (SBD), death domain, and C-terminal domain (24, 25). The protein plays an essential role in many cellular processes such as promoting the assembly of intercalated discs and axon initial segments, regulating voltage-gated Na^+^ and K^+^ channels, participating in lateral membrane biogenesis, and regulating E-cadherin endocytosis (26–29). A computational model of the SBD domain (982–1,462 aa, sequence identity 77.28%) is shown in Figure 2D. It is predicted that *ANK3* p.R1468H is at the end of the SBD (Figure 2F). This mutation affects evolutionarily conserved residues (Figure 2E). Although the variant is not in the highly conserved minimal region required for spectrin binding (surrounded by green lines in Figure 2D), Arg at the amino acid position 1,468 is altered to His, replacing a bulky side chain with a pentacyclic structure. This change may reduce clashes and affect the domain structure of the conserved sequence.

## Discussion

In this study, 20 variants and genes were filtered after WES and Sanger sequencing in three IA-aggregated families. We found that the variant *ANK3* c.4403G>A, p.R1468H (ENST00000280772, 44 exons) was shared in Pedigree 3 and Pedigree 1 (II-3). Venn analysis of the gene expression data revealed that *ANK3, GUCY1B3, FNDC1, SLC38A6*, and *ART3* were either upregulated or downregulated in the tissues of UIA compared to RIA and control samples. Furthermore, the variant *ANK3* c.4403G>A was significantly aggregated in IA cases (27 of 384 sporadic IA cases compared to 6 of 384 controls). Although the p. R1468H variation is not in the key part of the SBD [Figure 2D; (30)], the histidine at position 1,468 can introduce a 5-membered ring plane instead of a side chain as present in arginine, indicating that the variant alters the original domain structure. Variants of *ANK3* have not been reported previously to be associated with cerebrovascular diseases. Another variant, *GUCY1B3* c.17+5T>G, was found to be involved in angiogenesis, as it could affect the regulation of vascular endothelial growth factor to promote neovascularization (31, 32). However, the variant of *GUCY1B3* was not significantly associated with IA after Bonferroni correction.

*ANK3* encodes ankyrin-G protein in most mammalian cells (33). The MBD and SBD of ankyrin-G are highly conserved, but the carboxy-terminal regulatory domains are flexible to interact with the binding domains of membrane or spectrin and provide diverse functions (34). The MBD comprises 24 ANK repeats that are folded into a spatial structure of super-helical solenoid; this provides an ideal platform for membrane target arrangement (35, 36). Multiple families of proteins on the cell membrane can obtain ankyrin-binding activity, including voltage-gated Na^+^ channels and Na^+^-Ca^+^ exchangers (33). The SBD, or more specifically, the 160-amino acid ZU5 motif in SBD, acts as a bridge for the interaction between the ankyrin-associated membrane-protein complex and spectrin/actin cytoskeleton. This domain is critical for multiple physiological functions (37). The death domain is highly conserved (38). A recent study reported that the C-terminal domain contains autoinhibitory segments (39). These segments interact with their MBD for autoregulation to bind with their partner in various cellular localization processes.

Genome-wide association studies have shown that *ANK3* plays a role in schizophrenia and bipolar disorder (40, 41). The underlying biological mechanism may involve ankyrin-G deficiency resulting in the dysfunction of voltage-gated sodium channels and damage of synapses formed by internuncial Purkinje neurons. This, in turn, may affect the direct input mediated by their axo-axonic synapses and the modulation of output (42). Ankyrin-G also plays a role in arrhythmia (43, 44). It was reported that in a case of Brugada syndrome with an *SCN5A* variant in the Na_v_1.5 motif, the variant disrupted the interaction of Na_v_1.5 with ankyrin-G. Further studies suggested that the binding of ankyrin-G to βIV spectrin is essential for the function of calcium/calmodulin-dependent kinase II in the intercalated disc (45). This interaction can activate the phosphorylation of Na_v_1.5 to regulate I_Na_ (45, 46).

A recent study demonstrated that in *Ank3*+/– heterozygous mice, microtubule elongation was impaired, as the repression of *Ank3* decreased tubulin acetylation and enhanced the dynamics of microtubules causing microtubule instability (47). It is indispensable for microtubule activity to initiate and maintain the change in endothelial shape and dynamics of polarized cells induced by vascular endothelial growth factor (48). In addition, *Ank3* was shown to play a vital role in cadherin membrane localization and endocytosis in epithelial cells using *Ank3* conditional knockout mice (49). Cadherin-mediated cell adhesion and dynamics can affect cell migration as well as the regulation of angiogenesis by vascular endothelial growth factor and its signaling pathways (48). Thus, ANK3 may regulate the migration of vascular endothelial cells and affect cell–cell junctions, and variations in *ANK3* may weaken artery walls, thereby accelerating the formation of IA. However, further functional studies are needed to explore how *ANK3* variants mediate the progression of IA.

This study had several limitations. First, in WES, introns and non-coding sequences, which affect protein translation by altering DNA splicing or modifying transcriptional regulators such as enhancers, silencers and insulators, cannot be captured. Second, only 8 of 20 candidate variants were included in the replicate association studies in sporadic cases and controls. Considering that a limited number of variants can be selected in this procedure, online expression data and functional relevance were combined for further consideration. Statistical criteria were also strictly adjusted to minimize the rate of false-positives. However, apart from differentially expressed genes in IA tissues, a few genes expressed in circulating blood can also influence the vascular walls. Some variants only affect protein function but not gene expression in the cerebral arteries, but both of these can affect the occurrence and rupture of IA. Our current selection strategy of variants may lead to false-negatives. Thus, the remaining variants should also be evaluated in future studies. Third, the average age of IA-free control subjects was higher than that of familial and sporadic cases. However, considering the high paroxysmal age of IA to be between 45 and 60 years, and the peak incidence of aneurysmal SAH to be at 55 to 60 years of age, we recruited controls who were over 60 years old to avoid misclassification bias that an individual may become a patient with IA when he or she is older. Fourth, we did not perform functional studies; thus, the exact molecular mechanisms by which the variant functions in IA remain unclear. Further experimental studies are needed. Furthermore, although the *ANK3* variant has been identified in the Chinese population, it is unknown whether it is associated with IA in other ethnic populations, which should be further examined.

## Conclusions

Genetic studies showed that the candidate variant *ANK3* p.R1468H was significantly associated with IA (after Bonferroni correction). The variation p.R1468H was predicted to be present in the end of the ANK3 SBD, a highly conserved region, which may change the spatial structure and function of ankyrin-G. The *ANK3* variant may render cell–cell junctions vulnerable and modulate cell dynamics, thereby accelerating the formation and progression of IA. Further replicative studies and biological investigation of *ANK3* are warranted to clarify the underlying mechanism of *ANK3* in IA.

## Data Availability Statement

The datasets presented in this study can be found in online repositories. The names of the repository/repositories and accession number(s) can be found here: NCBI BioSample SAMN19471359, SAMN19471360, SAMN19471361, SAMN19471362, SAMN19471363, SAMN19471364, SAMN19471365, SAMN19471366, and SAMN19471367.

## Ethics Statement

The studies involving human participants were reviewed and approved by the Medical Ethics Committee of Clinical Pharmacology Institute, Central South University, China (CTXY-150002-1). The patients/participants provided their written informed consent to participate in this study.

## Author Contributions

JL, XL, and BL contributed to the data analysis. JZ, LX, SL, YL, DY, and CH contributed to the search and assessment of the available literature and data collection. JL, WJ, and JY wrote the manuscript and other authors helped to revise the text to the final form. All authors discussed and edited the manuscript.

## Conflict of Interest

The authors declare that the research was conducted in the absence of any commercial or financial relationships that could be construed as a potential conflict of interest.
