# Supplementary material for: A Rare Variant of ANK3 Is Associated With Intracranial Aneurysm
Source: Front Neurol. 2021 Jun 25;12:672570. doi: 10.3389/fneur.2021.672570 (PMC8267376; doi:10.3389/fneur.2021.672570)
Supplement: Supplementary file 1 [file Table_1.DOC]

**Table e-1. Primers design for Sanger sequencing.**

| **Gene** | **rs Number (dbSNP135)** | **Forward/Reverse** | **Primer** |
| --- | --- | --- | --- |
| *ANKRD36C* | rs76474100 | Forward | 5'-AATTAGCCAGGTGTGGTGGT-3' |
|  |  | Reverse | 5'-CAATCATAACCATCACCCCA-3' |
| *S100A1* | rs1046256 | Forward | 5'-CTCAGTGCTGTACCCTTCCC-3' |
|  |  | Reverse | 5'-AAGATGAGTTGCAGGCTTGG-3' |
| *PSPH* | rs75395437 | Forward | 5'-CCCAGAGGGCACTCTAAAGG-3' |
|  |  | Reverse | 5'-AACCTTGAGGACATCGGACC-3' |
| *MEGF6* | rs61910697 | Forward | 5'-CCCAGGACTGGCACAGGTAG-3' |
|  |  | Reverse | 5'-CACGGCCACTTCTGTGAGAG-3' |
| *PDSS1* | rs77826284 | Forward | 5'-AGGCGGAGGTTACAATGAGC-3 |
|  |  | Reverse | 5'-CCTCAGGCTCCCAGAATGCT-3' |
| *ANK3* | rs74777754 | Forward | 5'-TGTATGACAGGGTTTTCCTGC-3' |
|  |  | Reverse | 5'-GGGGTTACAAAATCCCAGTG-3' |
| *SLC38A6* | rs117560154 | Forward | 5'-TGGAGTTGTTCTTGAATGAGGA-3' |
|  |  | Reverse | 5'-GCCTCAAGAGCCACCACAGT-3' |
| *PPP1R37* | rs539710409 | Forward | 5'-GCTGCAAGCGCAACTTGGTG-3' |
|  |  | Reverse | 5'-CTGGTGGTAGGGAAGGTGAG-3' |
| *URB1* | rs148134142 | Forward | 5'-GGGGGAAGTGCAGGATGGTC-3' |
|  |  | Reverse | 5'-CACCACCACCCACTTCGTAA-3' |
| *MUC20* | rs2688539 | Forward | 5'-GGAGCTGGAATGACCACAGT-3' |
|  |  | Reverse | 5'-TCACCAGGGGCTTCAGCGAG-3' |
| *MYL5* | rs376244258 | Forward | 5'-GGGCTCAGGAAAGGAGAAGG-3' |
|  |  | Reverse | 5'-AGCTGGGATCCTGGGTTCGC-3' |
| *TMEM156* | rs140693293 | Forward | 5'-GGCCAAGGGATAAGCTATCA-3' |
|  |  | Reverse | 5'-TGGGGTGAGGAGTTGAGAGA-3' |
| *GUCY1B3* | rs76851701 | Forward | 5'-GAGGTCTTGGTATGTCGCCC-3' |
|  |  | Reverse | 5'-CTGTGCTATCACAGCTGCCA-3' |
| *FNDC1* | rs117546892 | Forward | 5'-AAGGAAAAACAGCGAGGCAT-3' |
|  |  | Reverse | 5'-TGACGTACTCCATTTGCCAT-3' |
| *YAE1D1* | rs79951226 | Forward | 5'-ACTTTTGTTCAGTGCTTTGCTC-3' |
|  |  | Reverse | 5'-TGTTCCAAAATCCATGTGGG-3' |
| *ZNF541* | rs140680651 | Forward | 5'-CGGGGGACCTGGCCTCTGGG-3' |
|  |  | Reverse | 5'-ACCAGGGTCCTACAGCAGAA-3' |
| *ZNF107* | rs375319415 | Forward | 5'-TGGCAAATCCTTTAACCAGTTC-3' |
|  |  | Reverse | 5'-AAAAGCTGTGCCACATTCTTTA-3' |
| *NLRP3* | rs117287351 | Forward | 5'-ACCTGGAGGATGTGGACTTG-3' |
|  |  | Reverse | 5'-AGGTGGAGGTTGCAGTGAGC-3' |
| *ADAD2* | rs191155110 | Forward | 5'-TTCTTGTTCCGGCAGCTCCT-3' |
|  |  | Reverse | 5'-CTTCAGCTGCCCGAGCACAT-3' |
| *PNMT* | rs60871117 | Forward | 5'-CATGTACAGCCAACATGCCT-3' |
|  |  | Reverse | 5'-GAAAGCTGGCAAGATCTGGG-3' |
| *ZNF446* | rs58632700 | Forward | 5'-GAAGCCACCACAAGGGGAGG-3' |
|  |  | Reverse | 5'-CACTCTGGCATCCCCTGCTG-3 |
| *ART3* | rs143599971 | Forward | 5'-CTATAGTGCATTGGGGCCTT-3' |
|  |  | Reverse | 5'-ATGCATTGGACAGTGGCTTG-3' |

Table e-2. Details of GEO databases for Venn analysis.

| **Accession Number** | **Authors** | **Year** | **Country** | **Samples (n)** | | **Differentially Expressed Genes (n)** | | | |
| --- | --- | --- | --- | --- | --- | --- | --- | --- | --- |
|  |  |  |  |  |  | **RIAvsUIA** | | **UIAvsControl** | |
|  |  |  |  | **IA** | **Control** | **Up-regulated** | **Down-regulated** | **Up-regulated** | **Down-regulated** |
| GSE 13353 | Kurki et al. | 2011 | Finland | RIA(11) +UIA(8) | - | 571 | 451 | - | - |
| GSE 15629 | Pera et al. | 2010 | Poland | RIA(8) +UIA(6) | MMA(5) | 177 | 46 | 322 | 266 |
| GSE 26969 | Li et al. | 2009 | China | UIA(3) | STA(3) | - | - | 61 | 1260 |
| GSE 54083 | Nakaoka et al. | 2014 | Japan | RIA(8) +UIA(5) | STA(10) | 132 | 66 | 940 | 593 |
| GSE 66238 | Bekelis et al. | 2016 | Lebanon | UIA(9) | STA(10) | - | - | 622 | 404 |
| RIA: ruptured intracranial aneusysm; UIA: unruptured intracranial aneurysm; MMA: middle meningeal artery; STA: superficial temporal artery. | | | | | | | | | |

Table e-3. Primers design for replication association study.

| **Gene** | **rs Number (dbSNP135)** | **Forward/Reverse** | **Primer** |
| --- | --- | --- | --- |
| *S100A1* | rs1046256 | Forward | 5'-ACGTTGGATGAATGTGGCTGTCTGCTCAAC-3' |
|  |  | Reverse | 5'-ACGTTGGATGGAAGGAGCTAGACGAGAATG-3' |
|  |  | Extended | 5'-CACAGTGGCCTGTAA-3' |
| *MEGF6* | rs61910697 | Forward | 5'-ACGTTGGATGAGACTCACCCAGGTTACAGG-3' |
|  |  | Reverse | 5'-ACGTTGGATGTGAGCCAGGTTCATTTGGAG-3' |
|  |  | Extended | 5'-gttgCCAGCGCTGTGACTGTGAC-3' |
| *PDSS1* | rs77826284 | Forward | 5'-ACGTTGGATGTTGCATGCTCGGGCCATTAG-3' |
|  |  | Reverse | 5'-ACGTTGGATGGATGCCATTGTGACACAGAG-3' |
|  |  | Extended | 5'-GGAAAGGGAAAGCCT-3' |
| *ANK3* | rs74777754 | Forward | 5'-ACGTTGGATGAGATGTCACAGGGAAGTTGG-3' |
|  |  | Reverse | 5'-ACGTTGGATGTTCCAGGCTCAGTCAAGTAG-3' |
|  |  | Extended | 5'-taTCAGTCAAGTAGCTGTAG-3' |
| *SLC38A6* | rs117560154 | Forward | 5'-ACGTTGGATGAGTGAGGTACCCAAAGAGTG-3' |
|  |  | Reverse | 5'-ACGTTGGATGTCGACCAGCTTAGGACATAC-3' |
|  |  | Extended | 5'-TTAGTCCTTCAAAGAAAAGAA-3' |
| *GUCY1B3* | rs76851701 | Forward | 5'-ACGTTGGATGCAGGCTTTACCAGCTAAATG-3' |
|  |  | Reverse | 5'-ACGTTGGATGCAAATTCCTGAGCTCAACGG-3' |
|  |  | Extended | 5'-ggacGCTTATGTGCTTTATGTGA-3' |
| *FNDC1* | rs117546892 | Forward | 5'-ACGTTGGATGTTACAGACAGTACACCGTGC-3' |
|  |  | Reverse | 5'-ACGTTGGATGTCACCCTGTGAAATACGGAC-3' |
|  |  | Extended | 5'-gaagCGCCTGTTAGCGAT-3' |
| *ART3* | rs143599971 | Forward | 5'-ACGTTGGATGGCTCAGCAGACAAGAGTTTG-3' |
|  |  | Reverse | 5'-ACGTTGGATGTCTGGCATGCCTATCAGTTG-3' |
|  |  | Extended | 5'-cattCCTATCAGTTGCTTACCAG-3' |

**Table e-4.** Results of Whole Exome Sequencing in 9 familial intracranial aneurysms.

| **Data** | **P1** | | | | **P2** | | **P3** | | |
| --- | --- | --- | --- | --- | --- | --- | --- | --- | --- |
|  | II2 | II3 | II5 | III2 | II2 | III1 | II1 | II3 | II5 |
| Sequencing and mapping data |  |  |  |  |  |  |  |  |  |
| Raw data yield (Gb) | 9.76 | 12.74 | 13.06 | 11.90 | 12.05 | 11.16 | 14.42 | 13.72 | 12.94 |
| No. of effective bases (Gb) mapped to genome | 9.47 | 12.39 | 12.67 | 11.54 | 11.66 | 10.81 | 13.23 | 12.63 | 10.74 |
| Exome capture |  |  |  |  |  |  |  |  |  |
| Effective bases for target region (Mb) | 5457 | 6960 | 7357 | 6605 | 6574 | 6199 | 7555 | 7181 | 6107 |
| Average sequencing depth on target region | 90.3 | 115.19 | 121.8 | 109.3 | 108.8 | 102.6 | 125 | 118.9 | 101.1 |
| Coverage of target region above 8 bases(%) | 97.7 | 98.7 | 98.7 | 98.4 | 98.5 | 98.4 | 98.7 | 98.6 | 98.3 |
| Fraction of target region covered in mapped reads (%) | 70.7 | 69.1 | 71.2 | 70.2 | 69.1 | 70.5 | 70.5 | 70.1 | 70.5 |
| SNV and InDel annotation |  |  |  |  |  |  |  |  |  |
| No. of total SNVs | 653710 | 811492 | 766319 | 751224 | 738711 | 703341 | 771406 | 808290 | 711830 |
| No. of total InDels | 83416 | 105085 | 97580 | 94701 | 90693 | 87252 | 96450 | 99060 | 82537 |
| SNVs, single nucleotide variants; InDels, insertion-deletion. | | | | | | | | | |
